# Behavioural and dopaminergic changes in double mutated human A30P*A53T alpha-synuclein transgenic mouse model of Parkinson´s disease

**DOI:** 10.1038/s41598-019-54034-z

**Published:** 2019-11-22

**Authors:** Tommi Kilpeläinen, Ulrika H. Julku, Reinis Svarcbahs, Timo T. Myöhänen

**Affiliations:** Division of Pharmacology and Pharmacotherapy/Drug Research Program, Faculty of Pharmacy, University of Helsinki, Helsinki, Finland

**Keywords:** Parkinson's disease, Parkinson's disease

## Abstract

Alpha-synuclein (aSyn) is the main component of Lewy bodies, the histopathological marker in Parkinson’s disease (PD), and point mutations and multiplications of the aSyn coding *SNCA* gene correlate with early onset PD. Therefore, various transgenic mouse models overexpressing native or point-mutated aSyn have been developed. Although these models show highly increased aSyn expression they rarely capture dopaminergic cell loss and show a behavioural phenotype only at old age, whereas *SNCA* mutations are risk factors for PD with earlier onset. The aim of our study was to re-characterize a transgenic mouse strain carrying both A30P and A53T mutated human aSyn. Our study revealed decreased locomotor activity for homozygous transgenic mice starting from 3 months of age which was different from previous studies with this mouse strain that had behavioural deficits starting only after 7–9 months. Additionally, we found a decreased amphetamine response in locomotor activity and decreased extracellular dopaminergic markers in the striatum and substantia nigra with significantly elevated levels of aSyn oligomers. In conclusion, homozygous transgenic A30P*A53T aSyn mice capture several phenotypes of PD with early onset and could be a useful tool for aSyn studies.

## Introduction

Parkinson’s disease (PD) is the second most common neurodegenerative disease after Alzheimer’s disease with an annual incidence of 160 per 100 000 people over 65 years old^1^. There are several risk factors including environmental and genetic factors associated with PD. However, less than 10% of PD cases have an identifiable genetic cause, thus development of the disease is most likely influenced by both complex genetic and environmental risk factors. PD is pathologically characterized by degeneration of dopaminergic neurons in the *substantia nigra pars compacta* (SNPc) and depletion of dopamine (DA) in striatal projections that leads to motor impairment^2–5^. Accumulation of alpha-synuclein (aSyn) in the brain and formation of filamentous inclusions called Lewy bodies and Lewy neurites are hallmarks of PD pathophysiology^6^. Inclusions of insoluble aSyn are also found in the brain of patients with Lewy body dementia and multiple system atrophy. The role of aSyn in the pathophysiology of PD was emphasized when mutations in the aSyn coding gene (*SNCA*) were found. The first discovered PD-related mutation in the *SNCA* gene was a point mutation where alanine in position 53 was substituted with threonine (A53T) leading to disruption in a helical formation^7^, and this mutation is associated with familial early onset PD^8^. Later, two more familial forms of early-onset PD associated with point mutations in the *SNCA*, namely A30P and E46K, were found^9,10^. Furthermore, duplications and triplications in the SNCA gene also increase incidence and severity of this disease^11,12^, further supporting the importance of aSyn in PD pathophysiology. aSyn aggregation leads to loss-of-function toxicity, and aggregates, particularly oligomers, damage several cellular organelles, while aSyn fibrils can propagate aSyn toxicity by cell-to-cell transfer^13^. Although, aSyn has been widely studied in the context and models of PD, its physiological role is more unclear. It has been suggested that aSyn interacts with DA transporter (DAT) which regulates the kinetics of extracellular DA in the synapses^14^. Aggregation of aSyn decreases the expression and membrane trafficking of DAT under normal circumstances indicating that aSyn has a role in DAergic neurotransmission^15,16^. Furthermore, it has been shown that aSyn colocalizes with SNARE proteins and aSyn aggregation redistributes them leading to decreased DA release in a mouse line expressing truncated aSyn^17,18^.

Transgenic (tg) mice overexpressing human aSyn have been a common tool to study aSyn toxicity and aSyn targeting drug therapies. Several mouse lines overexpressing wildtype (wt) aSyn or mutated forms of aSyn have been developed but these models mainly lack DAergic neuronal cell loss despite excessive aSyn expression (reviewed in^19^). Richfield *et al*.^20^ introduced a mouse model expressing double mutated human aSyn gene with both A30P and A53T point mutations under the rat tyrosine hydroxylase (TH) promoter (C57BL/6J-Tg(TH-SNCA*A30P*A53T)39Eric/J) that combined two well-characterized familial site mutations of SNCA to model PD. These tg mice expressed human aSyn in cell bodies, axons, and terminals in the nigrostriatal pathway, and had decreased locomotor activity at age 7–9 and 13–23 months, and lowered concentration of DA and its metabolites in the striatal tissue at age 16–18 months. Similar to several other aSyn tg mice, younger mice did not have significant changes in locomotor activity or in striatal DA concentration. However, both A30P and A53T familial point mutations in *SNCA* are a risk factor for early onset PD^19^ but these features were not captured in the earlier study. Therefore, the aim of this study was to breed a homozygous A30P*A53T aSyn tg mouse strain, and characterize if this animal model would capture the phenotype of early-onset PD. We designed PCR oligonucleotides and a new genotyping protocol to distinguish between wt, heterozygous, and homozygous animals in order to characterize behavioural and DAergic changes in homozygous A30P*A53T aSyn tg mice. Interestingly, we found several behavioural and histological changes that were not described in the original publication.

## Results

### A30P*A53T aSyn tg mice have altered locomotor activity

**Figure 1:**
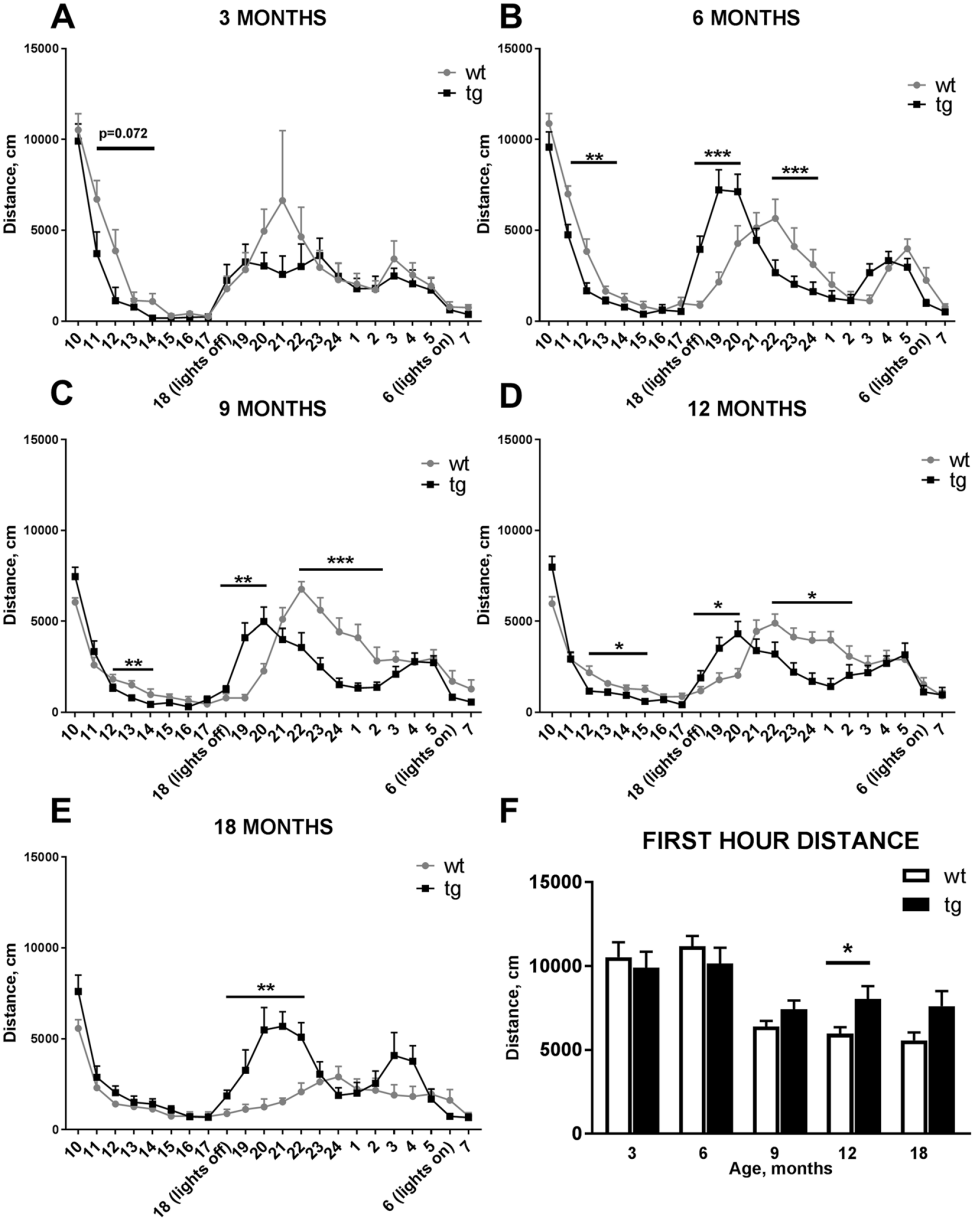
22 hour locomotor activity was altered between aSyn transgenic (tg) and wild type (wt) littermates in all age groups. At 3 months, significant changes between tg and wt animals were not seen (**A**). 6, 9, and 12 months old tg mice were more active compared to wt littermates right after the lights were turned off but less active than wt mice between 22:00 and 1:00 (**B–D**). 18 months old tg mice were more active during the dark time compared to wt littermates (**E**). When comparing locomotor activity during the first hour of the locomotor experiment, tg mice showed increased activity only at the age of 12 months (**F**). Data are expressed as mean ± SEM, n = 7–23. Repeated measures 2-way-ANOVA, (**A–E**); Student’s t-test (**F**); *p < 0.05, **p < 0.005, ***p < 0.001.

22-hour locomotor activity measurements showed differences between C57BL/6J-Tg(TH-SNCA*A30P*A53T)39Eric/J (tg) and wt littermates in all age groups (Fig. 1A–F). 3 months old mice did not have statistically significant alteration in overall locomotor activity. However, between the second and fifth hour (11–14), there was a trend that tg mice are less active compared to wt littermates (Fig. 1A, genotype effect: F_1,16_ = 3.705, *p* = 0.072, repeated measures 2-way ANOVA). 6 months old mice had statistically significant differences in locomotor activity in three different time intervals. Between 12:00 and 14:00, and 22:00 and 00:00 tg mice exhibited lower activity compared to wt littermates (Fig. 1B, 12:00–14:00 genotype effect: F_1,26_ = 9.982, *p* = 0.004; 22:00–00:00 genotype effect: F_1,26_ = 12.986, *p* = 0.001, repeated measures 2-way ANOVA). However, between 17:00 and 20:00 tg mice showed higher locomotor activity compared to wt littermates (Fig. 1B, genotype effect: F_1,26_ = 10.622, *p* = 0.003, repeated measures 2-way ANOVA). 9 months old tg mice had significantly lower activity between 12:00 and 14:00, and between 22:00 and 02:00 compared to wt littermates (Fig. 1C, 12:00–14:00 genotype effect: F_1,28_ = 9.725, *p* = 0.004; 22:00–02:00 genotype effect: F_1,28_ = 19.213, *p* = 0.0001, repeated measures 2-way ANOVA), while between 18:00 and 20:00 tg mice were more active compared to wt littermates (Fig. 1C, genotype effect: F_1,28_ = 8.924, *p* = 0.006, repeated measures 2-way ANOVA). Th effect was similar but more pronounced than the one seen in the 6 months old tg animals. 12 months old tg mice were more active compared to wt littermates during 18:00 and 20:00 period (Fig. 1D, genotype effect: F_1,35_ = 6.620, *p* = 0.014, repeated measures 2-way ANOVA). However, between 12:00 and 15:00, and between 22:00 and 02:00 tg mice had decreased activity compared to wt littermates (Fig. 1D, 12:00–15:00 genotype effect: F_1,35_ = 5.911, *p* = 0.029; 22:00-02:00 genotype effect: F_1,35_ = 6.540, *p* = 0.015, repeated measures 2-way ANOVA). At 6, 9 and 12 months, wt mice had delayed and higher horizontal locomotor activity after lights were turned off compared to tg mice (Fig. 1B–D). Interestingly, 18 months old tg mice were more active during the dark time between 18:00 and 22:00 (genotype effect: F_1,14_ = 20.753, *p* = 0.0004, repeated measures 2-way ANOVA). Total distance travelled during the first hour of the experiment did not show any differences between wt and tg groups except at 12 months the tg animals were more active during the first hour (Fig. 1F, t = 2.478, *p* = 0.023, Student’s t-test).

**Figure 2:**
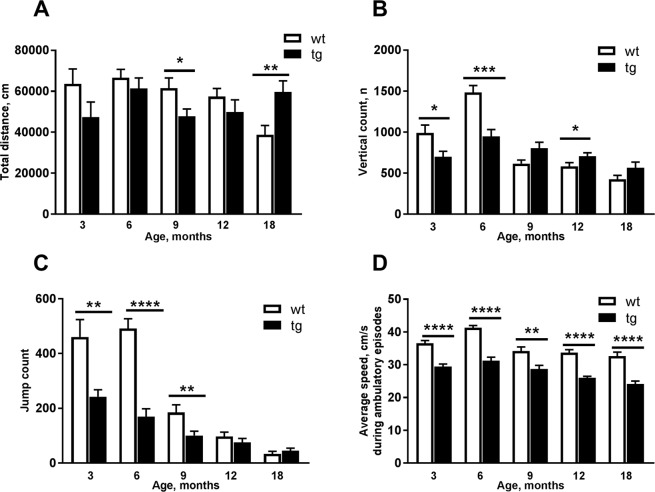
Comparison of total distance travelled, vertical counts, jump count, and average speed during 22 h locomotor activity measurements between aSyn transgenic mice (tg) and wild type littermates (wt). Total distance travelled was decreased in 9 months and increased in 18 months old tg mice compared to wt mice (**A**). 3 and 6 months old tg mice showed significantly less vertical counts compared to littermates (B). 12 old months tg mice had more vertical counts compared to wt mice (**B**). Jump counts were significantly lower in 3, 6, and 9 months old tg mice compared to wt littermates (**C**). Average speed of tg mice was significantly lowered in all age groups (**D**). Data are expressed as mean ± SEM, n = 7–23. Student’s t-test, *p < 0.05, **p < 0.005, ***p < 0.001, ****p < 0.0001.

**Figure 3:**
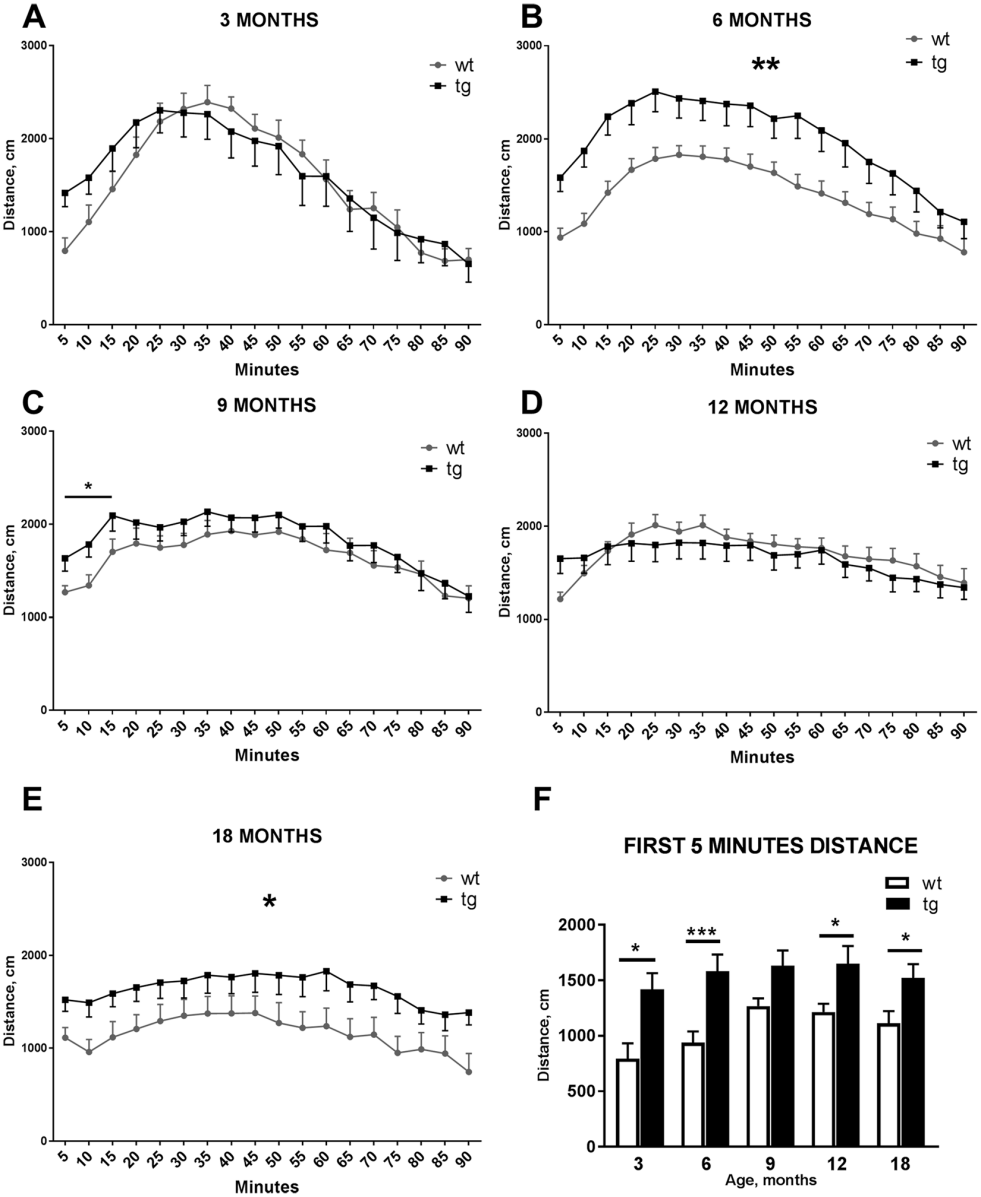
Amphetamine-induced locomotor activity was increased in the aSyn transgenic animals (tg) compared to wild-type (wt) littermates. After amphetamine administration, increased activity in tg mice was seen at the 6 and 18 month time-points, while only initial locomotor activity was increased in 9 months old tg animals (**A–E**). Total travelled distance of tg mice was increased in all age groups during the initial 5 minutes of the locomotor activity test compared to wt littermates (**F**). Data are expressed as mean ± SEM; n = 7–23. Repeated measures two-way ANOVA(**A–E**), Student’s t-test (**F**); *p < 0.05, **p < 0.01, ***p < 0.001.

During the 22-hour locomotor activity monitoring, differences were found between tg and wt mice in total distance travelled, vertical count, jump count, and average speed (Fig. 2A–D). Total distance travelled was significantly lower in 9 (Fig. 2A t = 2.28, *p* = 0.0301, Student’s t-test) and significantly increased in 18 (Fig. 2A t = 2.996, *p* = 0.0096, Student’s t-test) months old tg mice compared to wt mice. Vertical counts was already significantly lower in 3 (Fig. 2B, t = 2.54, *p* = 0.0218, Student’s t-test) and 6 (Fig. 2B, t = 4.409, *p* = 0.0002, Student’s t-test) months old tg mice. However, 12 months old tg mice had more vertical counts compared to wt littermates (Fig. 2B, t = 2.068, *p* = 0.045, Student’s t-test). Tg mice also had a significantly lower jump count at 3 (Fig. 2C, t = 3.835, *p* = 0.0013, Student’s t-test), 6 (Fig. 2C, t = 7.062, *p* < 0.0001, Student’s t-test) and 9 months (Fig. 2C, t = 2.785, *p* = 0.0093, Student’s t-test). Additionally, tg mice had a significantly lower average speed during the ambulatory episodes in all age groups (Figs. 2D, 3 (t = 6.028), 6 (t = 7.356), 12 (t = 8.311) and 18 (t = 5.718) months *p* < 0.0001; 9 months, t = 3.328, *p* = 0.0023, Student’s t-test).

### A30P*A53T aSyn tg mice have altered amphetamine-induced locomotor activity

90 min amphetamine-induced locomotor activity was assessed to evaluate differences in response to systemic amphetamine administration. No differences were observed between tg and wt mice at 3 and 12 months while at the 9 month time-point tg animals were more active compared to wt littermates only during the first 15 minutes after amphetamine administration (Fig. 3C, genotype effect: F_1,30_ = 4.790, *p* = 0.037, repeated measures 2-way ANOVA). However, 6 months old tg animals were significantly more active during the whole 90 minute period compared to wt littermates (Fig. 3B, genotype effect: F_1,32_ = 9.649, *p* = 0.004, repeated measures 2-way ANOVA). In 18 months old mice, a similar effect was observed (Fig. 3E, genotype effect: F_1,15_ = 4.632, *p* = 0.047, repeated measures 2-way ANOVA). In addition, total traveled distance during the first 5 minutes was significantly increased in 3 (Fig. 3F, t = 2.879, *p* = 0.01, Student’s t-test), 6 (Fig. 3F, t = 3.701, *p* = 0.0008, Student’s t-test), 12 (t = 2.306, *p* = 0.026, Student’s t-test) and 18 months (Fig. 3F, t = 2.429, *p* = 0.028, Student’s t-test) old tg mice compared to wt littermates. A similar effect was also observed in the 22-hour locomotor activity recordings but the effect was more noticeable in amphetamine-induced locomotor activity.

### Microdialysis and HPLC analysis of the nigrostriatal pathway of A30P*A53T aSyn tg mouse

**Figure 4:**
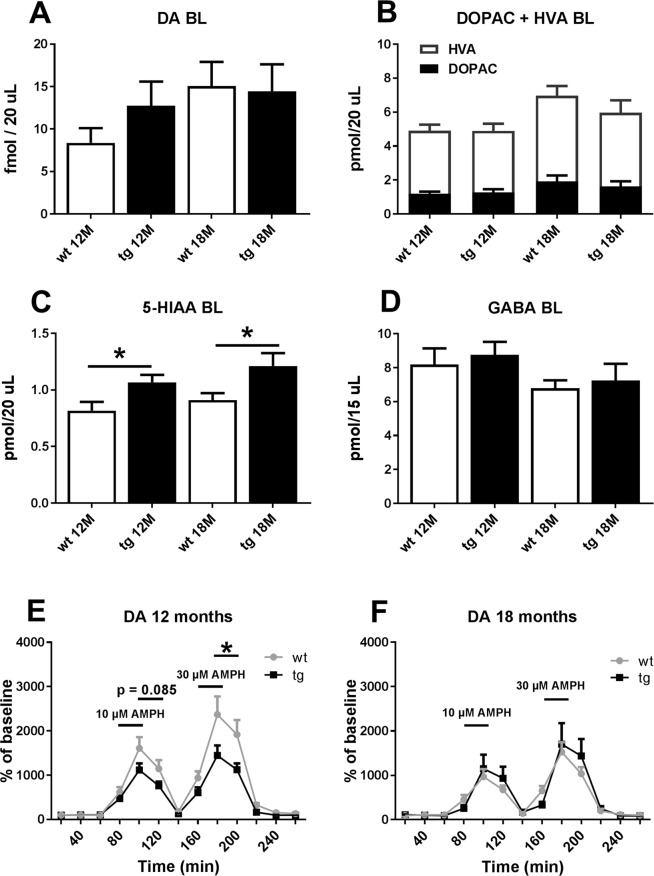
Striatal extracellular concentrations of dopamine (DA), its main metabolites DOPAC and HVA, 5-HIAA, and GABA were measured in the 12 months old and 18 months old wildtype (wt) and aSyn transgenic (tg) mice by microdialysis. Baseline level of DA (**A**), its metabolites (**B**), or GABA (**D**) were not changed but 5-HIAA was increased in both 12 months old and 18 months old tg mice (**C**). Amphetamine-induced increase in extracellular dopamine was lowered in the 12 months old tg mice with 30 µM d-amphetamine sulphate (AMPH) and there was a similar trend with 10 µM AMPH (**E**) but there was no difference between 18 months old mice (**F**). Data are expressed as mean ± SEM; 12 months: n = 10–12/group, 18 months: n = 7–8/group. Student’s t-test (A-D), repeated measures ANOVA (E-F); *p < 0.05.

The impact of the mutant aSyn transgene in the striatal DAergic function was studied in 12 and 18 month old tg mice and their wt littermates by microdialysis and tissue high-performance liquid chromatography (HPLC) analysis. In microdialysis, baseline level of extracellular striatal DA, its metabolites dihydroxyphenylacetic acid (DOPAC) and homovanillic acid (HVA), or gamma-aminobutyric acid (GABA) were not changed statistically significantly in the 12 months old mice (Fig. 4, DA: p = 0.224; DOPAC + HVA: p = 0.322; GABA: p = 0.637, Student’s t-test) or in the 18 months old mice (Fig. 4, DA: p = 0.884; DOPAC + HVA: p = 0.901, GABA: p = 0.668 Student’s t-test). Extracellular concentration of 5-hydroxyindoleacetic acid (5-HIAA) was increased in the 12 and 18-month old tg mice (Fig. 4C, 12-months, t = 2.467, p = 0.023; 18-months, t = 2.396, p = 0.032, Student’s t-test). Amphetamine-induced DA release elevated striatal extracellular DA concentration less in 12-month old tg mice than in wt littermates with 30 µM d-amphetamine sulphate concentration (Fig. 4E, F_1, 20_ = 4.988, p = 0.037, repeated measures ANOVA), and there was a similar trend with 10 µM d-amphetamine sulphate (Fig. 4E, F_1, 20_ = 3.286, p = 0.085, repeated measures ANOVA). A similar difference was not observed in the 18-month group (Fig. 4F, 10 µM d-amphetamine sulphate: F_1, 12_ = 0.573, p = 0.463; 30 µM d-amphetamine sulphate: F_1, 12_ = 0.470, p = 0.506, repeated measures ANOVA).

**Figure 5:**
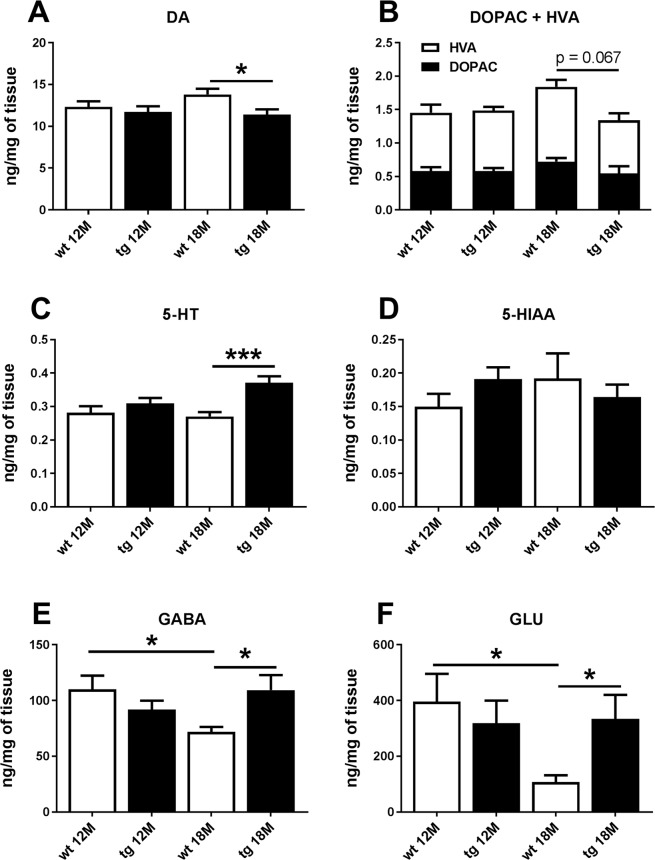
18 months old aSyn transgenic (tg) animals showed changes in DA, its metabolites, 5-HT, GABA and glutamate in the striatum. Striatal tissue concentrations of neurotransmitters and their metabolites were measured in the 12 months and 18 months old tg mice and their wild-type (wt) littermates. There was no alteration in neurotransmitters or their metabolites in the 12 months old mice (**A–F**). Dopamine was decreased in the striatum of 18 months old tg mice compared to wt littermates (**A**) and there was a similar trend in the dopamine metabolites (**B**). 5-HT (**C**), GABA (**E**) and glutamate (**F**) levels were increased in 18 months old tg mice but 5-HIAA was not changed (**D**). GABA (E) and GLU (F) had an age-dependent decrease in Wt mice but a similar phenomenon was not observed in other neurotransmitters in tg mice. Data are expressed as mean ± SEM; 12 months: n = 10–12/group, 18 months: n = 8/group. Student’s t-test, *p < 0.05; ***p < 0.001.

HPLC analysis revealed that striatal tissue concentrations of DA (Fig. 5A, p = 0.528, Student’s t-test), its metabolites DOPAC and HVA (Fig. 5B, p = 0.850, Student’s t-test), 5-HT (Fig. 5C, p = 0.273, Student’s t-test), its metabolite 5-HIAA (Fig. 5D, p = 0.127, Student’s t-test), GABA (Fig. 5E, p = 0.218, Student’s t-test) and glutamate (Fig. 5F, p = 0.553, Student’s t-test) were not significantly altered in the 12 months old mice. Striatal concentration of DA was lower in the 18 months old tg mice compared to wt littermates (Fig. 5A, t = 2.639, p = 0.019, Student’s t-test) and there was a similar trend seen for the DA metabolites (Fig. 5B, p = 0.067, Student’s t-test). Additionally, 5-HT (Fig. 5C, t = 4.283, p = 0.0008, Student’s t-test), GABA (Fig. 5E, t = 2.631, p = 0.020, Student’s t-test), and glutamate (Fig. 5F, t = 2.546, p = 0.023, Student’s t-test) were significantly elevated in the 18 months old tg mice. Striatal tissue concentration of 5-HIAA was not changed in the 18 months old mice (Fig. 5D, p = 0.520, Student’s t-test). Striatal GABA and glutamate were decreased in 18 months old wt mice compared to 12 months old wt mice (Fig. 5E, GABA: t = 2.647, p = 0.017; Fig. 5F, GLU: t = 2.508, p = 0.023, Student’s t-test), but a similar phenomenon was not observed in the tg mice.

### A30P*A53T aSyn tg mice have reduced TH immunoreactivity and increased aSyn oligomer-specific staining in the SNpc and striatum

**Figure 6:**
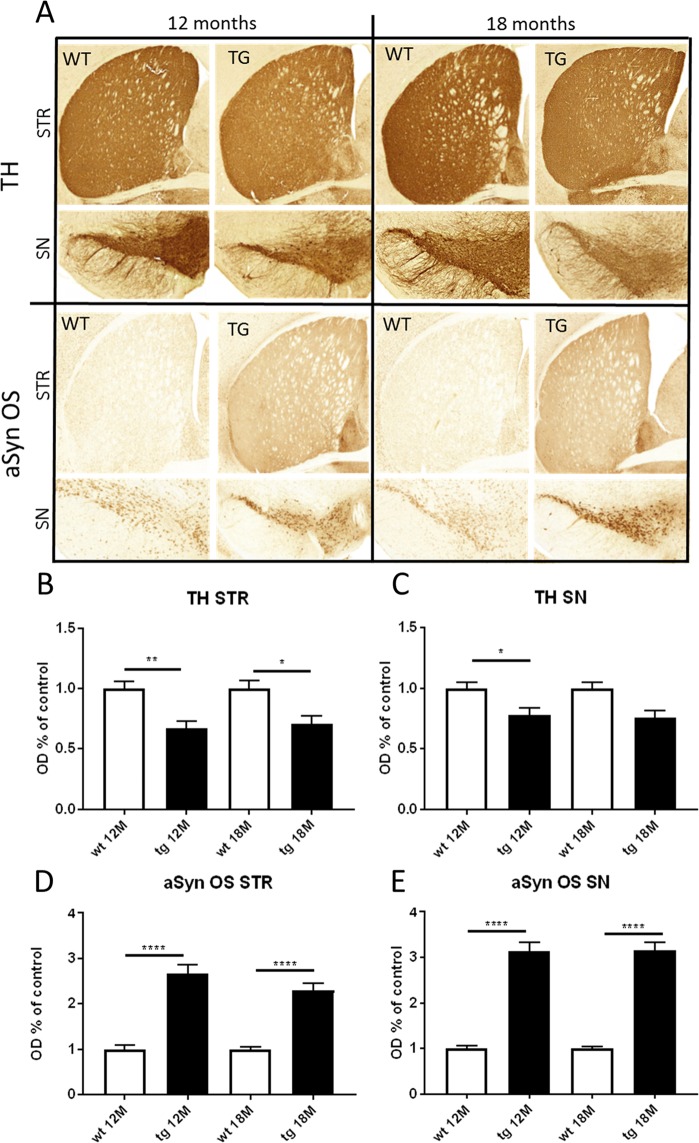
aSyn transgenic (tg) mice had significantly decreased TH and increased oligomer specific aSyn (aSyn OS) immunoreactivity in the striatum (STR) and substantia nigra (SN). Representative images of TH and aSyn OS staining from the striatum and substantia nigra of 12 and 18 month old wt and tg mice (**A**). TH optical density (OD) was significantly decreased in the STR and SN of 12 and 18 month old tg mice (**B,C**). OD of aSyn OS was significantly increased in both STR and SN at 12 and 18 months **(D,E**). Data are expressed as mean + SEM; n = 8–18. Student’s t-test,* p < 0.05, **p < 0.005, ****p < 0.0001.

Optical density (OD) analyses of TH and aSyn oligomer-specific immunoreactivity was quantified in the nigrostriatal tract of wt and tg mice (Fig. 6A–E). OD analyses revealed significant differences in the TH immunoreactivity in the striatum and SNpc. Both 12 and 18 months old tg mice had decreased TH immunoreactivity in both striatum (Fig. 6B, 12 months: t = 3.625, *p* = 0.0012; 18 months: t = 3.139, *p* = 0.0072, Student’s t-test) and SNpc (Fig. 6C, 12 months: t = 2.499, *p* = 0.019; 18 months: t = 3.175, *p* = 0.0067, Student’s t-test) compared to wt littermates. Accumulation of aSyn oligomers was identified by immunohistochemistry (IHC) in the SNpc and striatum. Both 12 and 18 months old tg mice had significantly increased immunoreactivity for aSyn oligomers in striatum (immunostained by aSynO5 antibody; Fig. 6A,D, 12 months: t = 6.36, *p* < 0.0001; 18 months: t = 7.716, *p* < 0.0001) and in SNpc (immunostained by aSynO5 antibody; Fig. 6A,E, 12 months: t = 8.084, *p* < 0.0001; 18 months: t = 11.53, *p* < 0.0001). OD analysis for total aSyn in the striatum revealed no significant differences in immunoreactivities in 12 and 18 months old tg mice compared to wt mice (see Supplementary Fig. S1).

## Discussion

In this study, our aim was to perform additional behavioural and biochemical characterization of the homozygous C57BL/6J-Tg(TH-SNCA*A30P*A53T)39Eric/J tg mice line and establish whether the mouse strain generates aSyn aggregate accumulation in the nigrostriatal tract. Our results show that homozygous tg mice had significant differences in locomotor activity in all age groups starting from 3 month-old animals, altered amphetamine response, and increased aSyn oligomer levels and decreased TH immunostaining in the nigrostriatal pathway. In an earlier study by Richfield *et al*.^20^ with the same mouse strain, the tg mice had age-dependent changes in locomotor activity starting from 7 months of age, significantly later than in our study. Therefore, we propose that a genotyping protocol that was able to separate heterozygous and homozygous mice was needed to generate homozygous animals.

Our detailed analysis of locomotor activity revealed that tg mice had decreased dark time activity but their highest activity period was shifted earlier compared to wt mice indicating abnormal locomotor behaviour. Interestingly, the most distinct differences in vertical and jump counts were observed in 3 and 6 months old animals, which could be considered to model motor symptoms of early-onset PD. However, at 12 months of age, the difference was not as clear as at earlier time points, and at 18 months tg mice were hyperactive in the locomotor test compared to wt littermates. This has been described earlier with A53T-aSyn overexpression tg mice strains that have shown to exhibit hyperactivity and anxiety, explained by alterations in function of DAT and an increased amount of D1 receptors^21–23^. Additionally, we observed significantly elevated glutamate levels in the striatal tissue of 18 months old tg mice compared to wt littermates which could partly explain the hyperactivity. It has been reported that the C57BL/6 mice strain has reduced motor activity at 12 and 22 months of age compared to 3 months old animals^24^, supporting reduced motor activity seen in 18 months old wt animals.

Richfield´s study (2002)^20^ demonstrated a reduced locomotor response to amphetamine in tg mice, however our study was contrary since 6, 9, and 18 months old tg mice had an increased response to amphetamine in the locomotor activity assay compared to wt littermates. Increased amphetamine-induced locomotor activity and decreased amphetamine-induced DA release in tg mice can indicate dysfunction of DAT. Interestingly, elevated amphetamine-induced DA release was only observed in 12 months old tg mice compared to wt littermates but not in 18 months old tg mice. This can be explained by the finding that the wt mice had decreased amphetamine-induced DA release at 18 months old compared to 12 months old wt mice, indicating that age-related changes in DAergic function in wt mice could cover the difference that was observed between the 12 months old wt and tg mice. This promotes the role of aSyn aggregation and toxicity in this mouse model since aSyn is known to regulate the function of DAT. In its normal state, aSyn is considered as a negative regulator of DAT and aSyn overexpression has been shown to modify basal and amphetamine-induced DA efflux^16^.

The earlier study also reported that striatal tissue concentrations of DA, DOPAC, and HVA were declined in 16–18 months old tg mice compared to wt littermates^20^. This was consistent with our study as the same effect was seen for 18 months old mice. Striatal DA was not yet changed in 12 months old tg mice compared to wt littermates although striatal and nigral TH were already decreased at 12 months. However, impairment in DAergic function of 12 months old mice was also observed in the microdialysis experiment as amphetamine-induced DA release was decreased and behaviour altered in tg mice compared to wt mice. Previous studies have shown that aSyn binds to TH^25^ and A53T mutated aSyn and aSyn aggregation and phosphorylation abolishes aSyn’s impact on TH^26,27^. Therefore, the elevated level of aSyn oligomers seen in tg mice could possibly explain reduced TH. Additionally, striatal tissue concentrations of 5-HT and GABA were increased which may arise from decreased DA regulation^28^. As a further support for this, striatal microdialysis revealed elevated baseline levels of 5-HIAA in tg mice compared to wt mice indicating increased 5-HT metabolism. Age-dependent decreases in striatal GABA and glutamate concentrations were observed in wt mice but not in tg mice. Similar findings in wt mice have been reported earlier by^29^. Decreased striatal DA can increase GABA and glutamate since DA downregulates striatal GABA and glutamate via DA receptor D2^28,30,31^. Additionally, accumulation of aSyn has been reported to induce enlargement of glutamatergic nerve terminals in the mouse striatum^32^. In conclusion, decreased DAergic function combined with the effect of aSyn on glutamatergic neurons is probably causing the difference in striatal GABA and glutamate between 18 months old wt and tg mice.

After aSyn aggregation was revealed as a potential key player in PD pathophysiology, several tg mouse lines overexpressing human aSyn with a A30P or A53T point mutation have been described. Mice expressing A30P aSyn have, in several studies, failed to show differences in locomotor activity, and in DA and TH levels despite accumulation of aSyn in several brain regions^33–35^. A53T mutant aSyn expressing mice usually have more severe motor impairments starting at older age, but malfunction of the DAergic system has not been clearly determined^36–38^. This has been one of the major problems when using aSyn tg mice since they do not model the most important feature in PD, the degeneration of DAergic system, very effectively. Additionally, these aSyn point mutations are a risk factor for early onset PD which occurs before the age of 40 to 50 years in humans^39^, and generally behavioural deficits are seen only in aged tg animals (>12 months). Our study demonstrated that A30P*A53T aSyn tg mice did not only have early behavioural changes but also changes in their DAergic system and a decreased amount of TH positive cells compared to wt littermates. These effects are most likely caused by toxicity from increased aSyn oligomers^40^ that we reported here in 12- and 18-month age groups. Behavioural changes indicate that aSyn toxicity starts earlier since locomotor deficits were already observed in 3 months old tg mice, and another double mutant A30P*A53T aSyn mouse line with a different promoter (Thy-1 promoter) described motor impairment starting from 3 months^41^, similar to our findings. Although A30P and A53T double mutation in *SNCA* has not been described clinically, our results indicate that this models early onset PD better than other tg mouse models.

In conclusion, there is still a lack of a mouse model for PD that displays motor and non-motor deficits typical for PD, alterations in the DAergic system and DAergic cell loss together with aSyn propagation and formation of aSyn-rich Lewy bodies. Such a research tool would be particularly critical when developing novel disease-modifying therapies targeting causes of PD. Our current study with homozygous double mutant A30P*A53T aSyn tg mice does not fulfil all of these requirements but it has early onset and age-dependent changes in locomotor activity and in the striatal DAergic function together with aSyn oligomer formation, and it could be a useful tool to model early onset PD with familial SNCA mutations.

## Methods

### Animals

Male C57BL/6J-Tg(TH-SNCA*A30P*A53T)39Eric/J (The Jackson Laboratory, USA) mice were housed under standard laboratory conditions (12 h light/dark cycle; room temperature, 23 ± 2 °C; relative humidity 50 ± 15%) in individually ventilated cages (Mouse IVC Green Line, Techniplast, Italy) with bedding (Aspen chips, 5 × 5 × 1 mm; 4HP, Tapvei, Estonia), nesting material (Aspen strips; PM90L, Tapvei), and Aspen brick (100 × 20 × 20 mm; Tapvei). Mice had access to chow food (Teklad 2016, Envigo, Huntingdon, UK) and filtered and irradiated water *ad libitum*. The experiments were performed according to European Communities Council Directive 86/609/EEC and were approved by the Finnish National Animal Experiment Board (ESAVI/441/04.10.07/2016).

### Genotyping

While we bred tg mice with wt mice to create homozygous mice, we found that homozygous male animals have a phenotypical feature where length of hair is much longer compared to the heterozygous and wt animals (Supplementary Fig. S2). Homozygous mice with the long-haired phenotypical feature and wt littermates were selected for sequencing. Sequencing service and genotyping primer design for the differentiation of the wt, heterozygous, and homozygous tg animals was provided by the University of Helsinki Genomic Core facility (Helsinki, Finland). Primer pairs provided were tested and all of the PCR products were sequence verified by the University of Helsinki Genomic Core facility. List of the best genotyping primer pairs and sequences can be found in Table 1. Routine DNA extraction and PCR were performed using REDExtract-N-Amp™Tissue PCR kit (#XNAT-1000RXN, Sigma-Aldrich, Saint Louis, MO, USA) with touchdown PCR cycling conditions provided in Table 2 and PCR product size was verified by agarose gel electrophoresis.

**Table 1:** Primer pairs and PCR product information for TH-SNCA*A30P*A53T genotyping.

Nr.	Primer sequences	PCR product, bp	Comments
1	Forward: 5′-ACAGCGAAAGTATCATTCATC-3′ Reverse: 5′-AACTGATAGAGGCTGTTTGG-3′	1190	Positive if construct has been inserted in chromosome 5 from position ~91496672→
2	Forward: 5′-ATCTTGGCTCACTGCAATC3′ Reverse: 5′-CTTCTCTCTAGTGTGCAAAGC-3′	849	Positive if construct has been inserted in chromosome 5 from position ← 91514836 ~
3	Forward: 5′-GTGTCACTTCTTAGCATAACG-3′ Reverse: 5′-CTTCTCTCTAGTGTGCAAAGC-3′	480	Positive if chromosome 5 as in original reference
4	Forward: 5′-ATCTTGGCTCACTGCAATC-3′ Reverse: 5′-AACTGATAGAGGCTGTTTGG-3′	1007	Positive if double construct of TH promoter and SNCA exists

**Table 2:** Cycling parameters used to genotype TH-SNCA*A30P*A53T mice.

	Step	Temperature (°C)	Time (min)
1.	Initial denaturation	94 °C	3 min
2.	Denaturation	94 °C	0:30 min
3.	Annealing	60 °C, −0,3 °C per cycle	0:30 min
4.	Extension	72 °C	1:30 min
		Go to step 2 33x times	
5.	Final extension	72 °C	10 min
6.	Hold	4 °C	∞

### Tissue processing

At the age of 12 or 18 months mice were sacrificed by cervical dislocation followed by dissection of the whole brain. The hemispheres were separated by using a brain matrix. The left hemispheres were frozen in isopentane on dry ice to collect striatal and nigral tissue samples for HPLC analysis as described in^42^ The right hemispheres were postfixed for 24 h in fresh 4% paraformaldehyde (PFA) at +4 °C and transferred to 10% sucrose in PBS (pH 7.4; 137 mM NaCl, 2.7 mM KCl, 10 mM Na_2_HPO_4_, 1.8 mM KH_2_PO_4_) overnight at +4 °C. On the next day, tissue was transferred to 30% sucrose solution in PBS until brains sank. Brains were frozen on dry ice and were kept at −80 °C until sectioning. Frozen brains were sectioned as 30 μm free-floating sections on a cryostat (Leica CM3050) and kept in a cryoprotectant solution (30% ethylene glycol and 30% glycerol in 0.5 M phosphate buffer).

### Immunohistochemistry (IHC)

Tyrosine hydroxylase (TH) IHC was done as described in^43^. In short, after blocking endogenous peroxidase activity sections were incubated for 30 min in 10% normal goat serum to block nonspecific binding, after which the sections were incubated overnight in rabbit anti-TH primary antibody (1:2000; AB152, RRID:AB_390204, Merck, Darmstadt, Germany). Subsequently, the sections were placed in goat anti-rabbit biotin-conjugated secondary antibodies (1:500; BA1000, RRID:AB_2313606, Vector Laboratories, Peterborough, UK). The signal was enhanced with the avidin– biotin complex method (Standard Vectastain ABC kit, RRID: AB_2336819, Vector Laboratories) and visualized with 3,3′-diaminobenzidine (DAB). Oligomer-specific -aSyn IHC was performed as described in^43^ using the Basic Vector Mouse on Mouse (M.O.M.) Immunodetection Kit (BMK-2202, RRID:AB_2336833, Vector Laboratories). In short, after blocking endogenous peroxidase activity, sections were incubated for 30 min in M.O.M. Mouse Ig Blocking Reagent to block nonspecific binding, and 5 min in M.O.M. diluent, and transferred overnight in mouse anti-human aSyn oligomer-specific primary antibody (1:200 in M.O.M. diluent; AS132718, RRID: AB_2629502, Agrisera, Vännäs, Sweden). The sections were then incubated with goat-anti-mouse HRP-conjugated secondary antibody (dilution, 1:300 in M.O.M. diluent, catalog #31430, RRID:AB_228307, Thermo Fisher Scientific, Grand Island, NY, USA) and visualized with DAB.

### Microscopy and optical density analyses

The optical densities (ODs) of TH and oligomer-specific aSyn from striatum and SNPc were determined as described earlier in^43^. Digital images were scanned at 40x magnification with a Pannoramic Flash II Scanner (3DHISTECH, Budapest, Hungary), and three coronal sections from each mouse were processed for further analyses with Pannoramic Viewer (version 1.15.3. RRID:SCR_014424, 3DHISTECH). Images were converted to grayscale and inverted, and line analysis tools for striatum or freehand for SN in ImageJ (1.48b; RRID:SCR_003070, NIH) was used to measure the OD of immunoreactivity. Corpus callosum was used to subtract the background optical density of each section and then normalized to the control mice.

### Behavioral assessments

Locomotor activity was measured every three months from 3 to 12 months and 18 months using automated open field locomotor activity chambers (Activity monitor, SOF-812, Med Associates inc, Georgia, USA). Total photobeam breaks were recorded for 22 h (starting at 10:00) for horizontal, vertical, and ambulatory movements. Amphetamine-induced locomotor activity was assessed every third month. Mice were habituated in locomotor boxes for 30 minutes before amphetamine (3 mg/kg i.p) was administered. Locomotor activity was measured for 90 minutes immediately after the amphetamine administration.

### Surgical procedures for microdialysis

Guide cannula (AT4.9.iC, AgnTho’s, Sweden) for microdialysis was inserted into the left striatum at 0.6 mm anterior, 1.8 mm lateral, and 2.7 mm below the dura (stereotaxic coordinates according to^44^ in a stereotaxic operation as described in Julku *et al*.^42^. The guide cannula was fastened to the skull with dental cement (Aqualox, Voco, Germany) and two stainless steel screws (1.2 × 3 mm, DIN84, Helsingin Ruuvihankinta, Finland). Mice were anesthetized with isoflurane (4% induction, 1.5–2.0% maintenance; Attane vet 1000 mg/g, Piramal Healthcare, UK). Buprenorphine (0.1 mg/kg s.c.; Temgesic 0.3 mg/mL, Reckitt Benckiser Healthcare, UK) was given before the operation and 5–6 h after the surgery, and carprofen (5 mg/kg s.c.; Norocarp vet 50 mg/mL, Norbrook Laboratories Ltd, Ireland) was given immediately after the surgery and 24 h after the surgery to relieve post-operative pain.

### Microdialysis

Microdialysis was performed in the 12 months old and 18 months old tg mice and their wt littermates as described earlier in^42^. Shortly, a microdialysis probe (1-mm cuprophan membrane, o.d. 0.2 mm, 6 kDa cut-off; AT4.9.1.Cu, AgnTho’s) was inserted into the guide cannula 2 h before the experiment, and the probe was perfused with a modified Ringer solution (147 mM NaCl, 1.2 mM CaCl_2_, 2.7 mM KCl, 1.0 mM MgCl_2_, and 0.04 mM ascorbic acid) at a flow rate of 2.0 μL/min. Four baseline samples were collected (20 min/40 μL/sample) after the stabilization period. After the collection of baseline samples, the probe was perfused 2 × 20 min with 10 μM and 30 μM d-amphetamine sulphate with 2 × 20 min recovery time between the concentrations. The concentrations of DA, its metabolites, DOPAC and HVA, and 5-HIAA as well as GABA in dialysates were measured using the HPLC methods that have been described earlier in^42^.

### Tissue HPLC analysis

Striatal tissue samples were punched below corpus callosum +0.74 mm from bregma to 2 mm depth by using sample corer (i.d. of 2 mm) with a plunger (Stoelting Co, Wood Dale, IL, USA) on a cryostat (Leica CM3050). Tissue processing was done as earlier described in^42^. The concentration of DA, its metabolites DOPAC and HVA, 5-HT, its metabolite 5-HIAA, GABA and glutamate in the tissue samples of striatum were analyzed with an HPLC as earlier described in^42^. The concentrations were calculated as nanograms per milligram of brain tissue.

## Supplementary information

Suppelementary information

## Data Availability

All materials, data and associated protocols are available to readers.
