# Supplementary material for: Behavioural and dopaminergic changes in double mutated human A30P*A53T alpha-synuclein transgenic mouse model of Parkinson´s disease
Source: Sci Rep. 2019 Nov 22;9:17382. doi: 10.1038/s41598-019-54034-z (PMC6874660; doi:10.1038/s41598-019-54034-z)
Supplement: Supplementary file 1 — Suppelementary information [file 41598_2019_54034_MOESM1_ESM.pdf]

Supplementary information for the manuscript “Behavioural and dopaminergic changes in double mutated human A30P*A53T alpha-synuclein transgenic mouse model of Parkinson´s disease”

by Tommi Kilpeläinen, Ulrika H. Julku, Reinis Svarcbahs, Timo T. Myöhänen

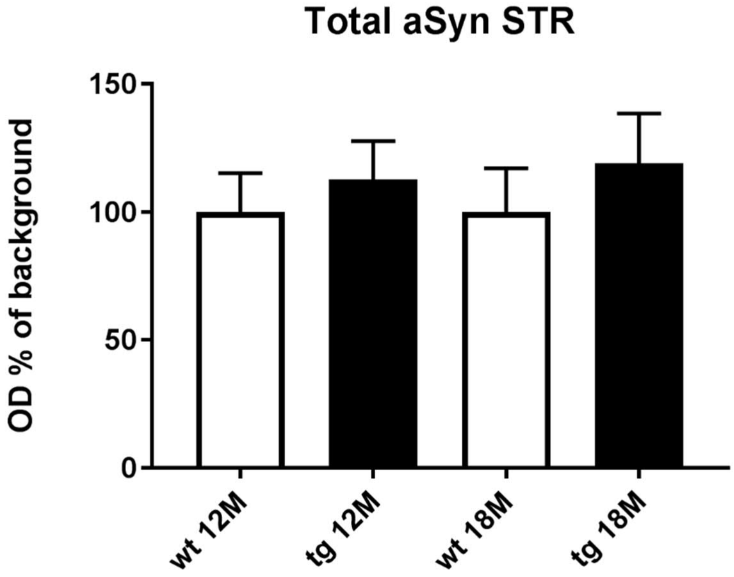

Figure S1. Total alpha-synuclein (aSyn) staining by using ab6162 (AbCam) from wild-type (wt) and transgenic (tg) mouse striatum did not reveal any statistical differences.

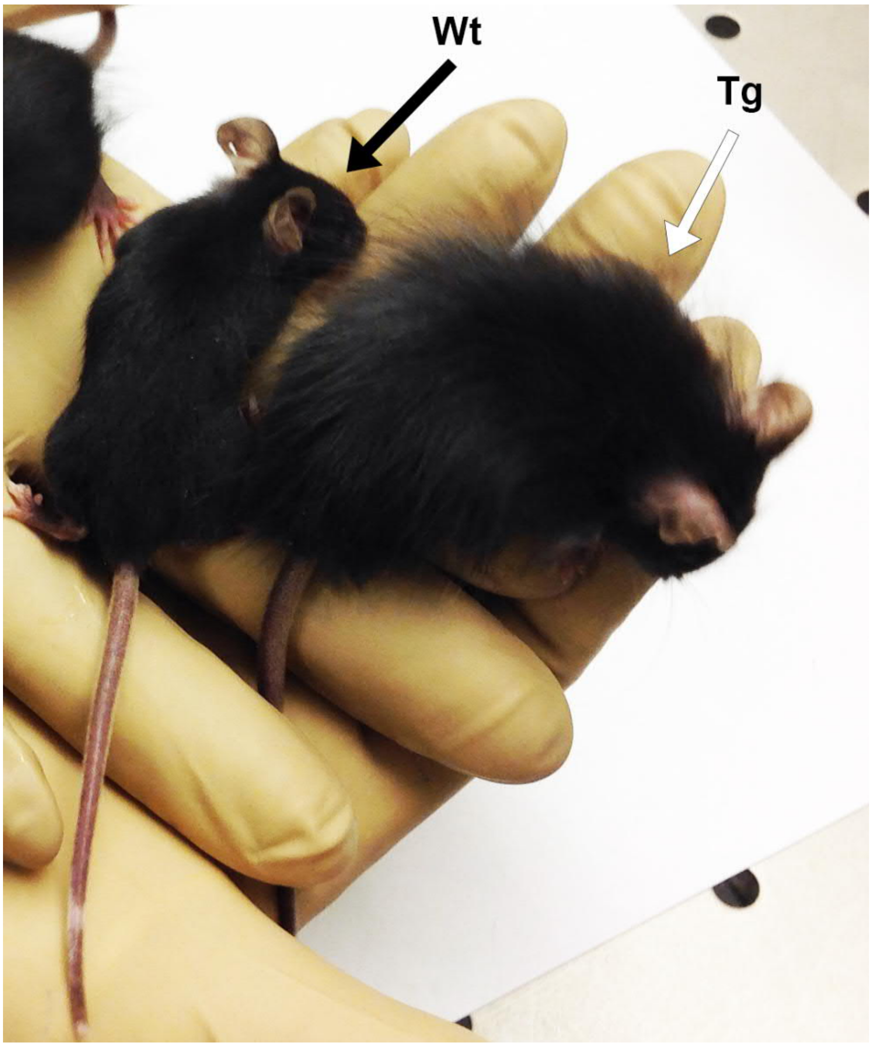

Figure S2. Homozygous A30P*A53T transgenic mice showed long-haired phenotype that was not seen with wild-type C57Bl6/Rcchsd mouse.
